# Can Corporate Social Responsibility Promote Employees’ Taking Charge? The Mediating Role of Thriving at Work and the Moderating Role of Task Significance

**DOI:** 10.3389/fpsyg.2020.613676

**Published:** 2021-01-28

**Authors:** Aimin Yan, Liping Tang, Yingchun Hao

**Affiliations:** Business School, Central South University, Changsha, China

**Keywords:** corporate social responsibility, thriving at work, task significance, taking charge, conservation of resource theory

## Abstract

There is growing evidence to suggest that employees’ perceptions of their employer’s corporate social responsibility (CSR) positively influences their attitude and behavior. An increasing number of scholars have called for further explorations of the microfoundations of CSR. To that end, this study takes the conservation of resources perspective to examine relationships and the perception of CSR by employees, considering areas such as thriving at work, task significance, and employees taking charge. By analyzing 444 questionnaires completed by employees in China and using the conditional process analysis to test a hypothesis, results showed that the association between employees’ CSR perception and taking charge is significantly and positively correlated, with thriving at work mediating the connection. We also found that task significance negatively moderates the mediating effect between CSR and taking charge, such that the lower the level of task significance of a job, the more positive the effect of CSR on taking charge via thriving at work. These findings have theoretical implications for micro-level CSR research and managerial implications for entrepreneurs.

## Introduction

In the spring of 2020, the COVID-19 virus swept across China, and enterprises from all over the nation donated spontaneously to Hubei Province, the hardest-hit area in the country. The benevolence of enterprises highlights their responsibilities as social citizens. Looking back into history, the concept of corporate social responsibility (CSR) was widely diffused in the 1960s (Wang et al., 2016). CSR refers to the organizational actions and policies taken by an enterprise in a specific context, and it is characterized by taking into account the expectations of stakeholders and the triple bottom line of economic, social, and environmental performance (Aguinis and Glavas, 2012). Typical CSR activities include supporting humanitarian causes, charitable donations, environmental protection, work-life balance, training, assistance programs, and so on (De Roeck et al., 2014). From the aforementioned definitions, we can see that in addition to contributing to society and the natural environment (Rupp and Mallory, 2015), CSR also acts as an important part of strategic human resource management, which can become a significant source of organizational competitive advantage (Gupta and Sharma, 2016). Moreover, with the improvement of corporate governance structure, the idea of CSR has been almost universally sanctioned and promoted by many enterprises and even incorporated into the development strategies of the enterprises (Wang et al., 2016).

Over the past few decades, there has been a significant increase in research on macro-level CSR (Lee, 2008), mainly focusing on the effect of CSR on the organizational strategy of enterprises (Gond et al., 2017), corporate governance (Ling et al., 2018), financial outcomes (Mcwilliams and Siegel, 2001; Aguinis and Glavas, 2012), and corporate image (Du et al., 2010). As for the micro-level research of CSR, despite the growing number of studies investigating the effects of CSR on employee behaviors (Rupp and Mallory, 2015), examining ideas such as organizational citizenship behavior (Rupp et al., 2013), pro-environmental behavior (Tian and Robertson, 2019), helping behavior (Wang et al., 2017), and employee engagement (Rupp et al., 2018), as well as employee creativity (Hur et al., 2018), there are still some research gaps in micro-level research on CSR (Glavas, 2016). More specifically, in contrast to the positive and affiliative organizational citizenship behaviors mentioned above (Burbano, 2016), we still know very little about the microfoundations of CSR in relation to challenging organizational citizenship behaviors such as taking charge behavior and voice behavior (Mcallister et al., 2007; Mackenzie et al., 2011; Gond et al., 2017). Although previous studies have indicated a positive relationship between CSR and the voice behavior of employees (Ilkhanizadeh and Karatepe, 2017; Wang et al., 2019), it is important for scholars to further explore the underlying mechanism between CSR perception and employee behaviors in terms of taking charge.

As a type of challenging organizational citizenship behavior (Mcallister et al., 2007), taking charge is defined as initiative behavior in employees, who make constructive efforts spontaneously to change the way things work. Previous research proposes that taking charge can play a crucial role in facilitating organizational innovation, enhancing organizational adaptability, and improving the long-term viability of firms (Tae-Yeol and Zhiqiang, 2017). Most previous studies discuss the antecedents of taking charge from the perspective of individual characteristics (Fuller and Marler, 2009; Love and Dustin, 2013; Baroudi et al., 2017) and leadership styles (Li et al., 2013b, 2016; Wang et al., 2020; Zeng et al., 2020b). Given that the leadership styles and individual characteristics are difficult to change in a short time, there is a more practical value to discussing the underlying mechanism of enterprise, how employees actively undertake responsibilities and take charge (Li et al., 2013a).

Several recent studies have explored the relationships between CSR perception and challenging organizational citizenship behaviors (e.g., employee voice behavior), primarily based on ethical climate theory (Wang et al., 2019), social exchange, and social information processing theory (Ilkhanizadeh and Karatepe, 2017). Although these findings have provided useful insights about the link between CSR and challenging organizational citizenship behaviors, there are still other underlying mechanisms that can explain the relationship between CSR perception and these behaviors (Aguinis and Glavas, 2012). We assumed that the relationships between CSR perception and employees’ taking charge (a type of challenging organizational citizenship behaviors) may be supported by the conservation of resource theory (COR). COR theory posits that individuals are always motivated to acquire, protect and retain their own resources (Hobfoll, 2001), and those with abundant resources are not easily affected by resource depletion and are more capable of acquiring more resources to realize the spiral of resource appreciation (Hobfoll, 2002). Enterprises actively undertake social responsibility to display a responsible, compassionate, and caring corporate image (Farooq et al., 2014), which acts as a contextual resource for employees (Brummelhuis and Bakker, 2012). Employee-focused CSR practices such as employee training programs and continuing education programs could also be regarded as a social support (Xanthopoulou et al., 2009) that can help employees get more resources and reduce concerns about the risks of taking charge (Halbesleben et al., 2014).

Moreover, according to the resource gain spiral—those who possess resources are more capable of gain, and that initial resource gain begets further gain (Hobfoll, 2001). We assume that employees working in responsible enterprises are easy to gain some contextual resources, and abundant initial resource accumulation can promote employees to obtain an increasing number of additional resources. Accordingly, this study introduces thriving at work as a mediator between CSR perception and employees’ taking charge. To be more specific, as an individual energy resource (Hobfoll, 2001), thriving at work refers to a psychological state in which an individual feels both vitality and learning at work (Spreitzer et al., 2005). It can not only help employees recover from resource depletion but also encourage them to improve knowledge and gain new skills, accumulating personal resources and further promoting the likelihood that they will take charge (Sonnentag and Fritz, 2007).

An increasing number of scholars call for the discussion of boundary conditions, which is more conducive to exploring the effectiveness of micro-level CSR (Vlachos et al., 2017). Through reviewing 268 micro-CSR papers, Gond et al. (2017) further argue that existing research on CSR boundary conditions mainly focused on factors such as personal characteristics and cultural differences, which are difficult to change for organizations. This study takes into account task significance from the perspective of work design as the boundary conditions that influence the relationship between employees’ CSR perception and taking charge. Task significance reflects the extent to which the job has a substantial impact on the lives and work of people inside or outside the organization (Hackman and Oldham, 1975). It can also be regarded as a structural resource according to COR theory (Brummelhuis and Bakker, 2012). Although task significance is an essential dimension of a job that allows employees to experience greater work meaningfulness (Grant, 2008), employees whose jobs are higher in task significance may need to spend more time and energy in completing tasks effectively, which is also a process of personal resource consumption (Hobfoll et al., 2018). Accordingly, we argue that task significance may negatively moderate the relationship between employees’ CSR perception and taking charge via thriving at work.

According to the suggestion of scholars such as Rupp et al. (2018) and Ng et al. (2019), this study focuses on employees’ perception of their employer’s CSR activities rather than objective organizational CSR actions. We aim to build a theoretical framework that provides more insight into the relationships between CSR perception and taking charge behavior. We see this work as making at least four novel contributions to research and practice. First of all, different from the previous micro-level CSR research, which mainly focuses on affiliative organizational citizenship behavior, the primary contribution of this study is to explore the relationship between employees’ CSR perception and taking charge (a type of challenging organizational citizenship behaviors), which will further broaden literature on outcome variables of micro-level CSR. Second, relying on the gain spiral of COR theory, this study posits CSR as a social support and contextual resource, and thriving at work as an individual energy resource to examine the relationships among employees’ CSR perception, thriving at work, and employees’ taking charge. It will not only promote the development of COR theory but also expand the theoretical discussion of the underlying mechanism between CSR perception and employees’ behavior. Third, by introducing task significance from the perspective of work characteristics, we assume that employees undertake important work as a process of individual resource depletion to explore the negatively moderating effect of task significance, and how the results of moderation analysis enrich discussion of the boundary conditions of micro-level CSR research. Last but not least, from a practical perspective, this study has several important implications for entrepreneurs and managers.

## Theoretical Background and Hypotheses

### CSR and Taking Charge

In the 1960s, the concept of corporate social responsibility (CSR)—“businesses bearing a responsibility to society and a broader set of stakeholders beyond its shareholders” gained currency (Wang et al., 2016). Strategic management scholars paid initial attention to CSR to explore its influence from the macro level. It was not until the last decade that micro-level CSR from the perspective of employees was brought to the forefront (Rupp and Mallory, 2015). Scholars have investigated the various behavioral outcomes of CSR (Gond et al., 2017), among which extra-role and affiliative organizational citizenship behaviors (OCBs) have received the most attention in this domain (Farooq et al., 2017). However, relatively little is known about the effects of CSR on challenging organizational citizenship behaviors. “Taking charge” is one of a number of challenging organizational citizenship behaviors (Morrison and Phelps, 1999; Mcallister et al., 2007). It is change-oriented and focuses on the internal means for accomplishing organizational goals, such as work methods, policies, and procedures (Morrison and Phelps, 1999). In addition, taking charge clearly has potential risks—it challenges and changes the *status quo*, which may cause conflicts at work (Fuller and Marler, 2009). What is worse, employees can suffer from damaged personal reputation if they fail to persuade their leaders or colleagues to view the initiative as appropriate or constructive (Morrison and Phelps, 1999; Parker and Collins, 2010).

Based on COR theory, there are three reasons why CSR can promote an employee taking charge. First, enterprises are motivated to engage in CSR activities such as environmental sustainability programs and philanthropic giving, which is conducive to gaining a good external reputation and establishing a good corporate image in society (Du et al., 2010). A good external reputation and corporate image are stable contextual resources that are valued by employees (Brummelhuis and Bakker, 2012). In this context, employees make more effort to maintain and accumulate this resource, such as taking charge of their work to help the enterprise in undertaking social responsibility. Second, employee-oriented CSR such as creating a safe working environment, formulating diversified policies, providing employees with training and continuing education opportunities, could be regarded as a resource of social support for employees (Xanthopoulou et al., 2009). Relying on the resource investment principle of COR theory, when enterprises provide large amounts of social support, employees will become more competent at their job than before, and become willing to make further investment to achieve the resource accumulation even if they are confronted with some risks (Hobfoll et al., 2018). In other words, enterprises engaged in CSR suggest to employees that the organization values a caring and fair management approach (Rupp et al., 2013; Jones et al., 2016), which are more likely to help employees in reducing their concerns about risks and taking charge. Third, existing research shows that work environment and characteristics are important factors affecting and challenging organizational citizenship behaviors (Ng and Feldman, 2012). As an organizational phenomenon (Wang et al., 2016), CSR can promote employees to have a voice, by mediating the role of organizational trust and organizational pride (Aimin et al., 2017, 2018). Taking charge behavior and voice behavior belong to challenging organizational citizenship behaviors (Mcallister et al., 2007; Mackenzie et al., 2011), both of which are constructive and risky. It provides lateral support for the hypothesis of this study. Consequently, the following hypothesis is proposed in this study:

Hypothesis 1 (H1). CSR is positively related to taking charge.

### The Mediating Role of Thriving at Work

Thriving at work is defined as the psychological state in which the individual experiences both a sense of vitality and learning at work. Vitality represents the emotional dimension, which means that an individual has energy and aliveness in his or her work, and it is a pleasant work experience and can be regarded as a state of activation of positive emotions (Porath et al., 2012). Learning represents the cognitive dimension, which refers to the psychological state of an individual who acquires and applies knowledge and skills at work (Spreitzer et al., 2005). Hobfoll (1989, 2002) defined resources as things such as objects, personal characteristics, conditions, or energies that are valued by the individual or that serve as a means for attaining these objects, personal characteristics, conditions, or energies. Thriving is an energy resource for individuals (Hobfoll, 2001; Brummelhuis and Bakker, 2012) because vitality can help individuals recover from resource depletion, while learning is conducive to the mastery and improvement of individual knowledge and skills, and increases the ability to obtain other resources (Sonnentag and Fritz, 2007).

Scholars recognize that structural features are starting points for understanding what enables people to thrive and develop at work (Spreitzer et al., 2005). As a type of contextual resources (Brummelhuis and Bakker, 2012), CSR is an organization’s discretionary initiative that aims to preserve and contribute to social welfare (Waldman et al., 2006; Barnett, 2007), which facilitates employees and enables them to hold positive attitudes toward the organization (De Roeck and Maon, 2018). According to COR theory, CSR can promote employees’ thriving at work through two paths: on the one hand, an organization actively undertakes social responsibilities and indicates that the organization is willing and able to dedicate resources to serve its stakeholders’ interests (Ng et al., 2019). Perception of organizational competence and responsibility can be treated as a contextual resource. Therefore, employees working in such kinds of organizations will experience the meaning of work, evoke their positive emotions, and increase the level of vitality (Shea and Hawn, 2019). On the other hand, CSR provides information about what the organization stands for and what they can expect in terms of personal treatment (Tourigny et al., 2019). For example, providing a safe working environment and opportunities for training and continuing education for employees’ career development reflects the fact that enterprise is responsible for employees (Farooq et al., 2017). The employee-focused CSR practices mentioned above can be regarded as a social support to provide opportunities for employees to learn (Walumbwa et al., 2018). Based on COR theory, structural contextual resources enable someone to avoid or solve contextual demands and to collect new resources (Brummelhuis and Bakker, 2012). That is to say, individuals who work in a responsible enterprise can be motivated to protect their current resources (social support and contextual resources that come with enterprise undertaking CSR) and acquire new resources (thriving at work) (Halbesleben et al., 2014). Accordingly, the following hypothesis is proposed:

Hypothesis 2 (H2). CSR is positively related to thriving at work.

According to COR theory, loss spiral and gain spiral are two main processes of the theory (Hobfoll, 1989). The loss spiral reflects a process whereby there is first a loss in personal resources due to contextual demands, which induces further loss, and the gain spiral indicates that individuals with more resources are not easily affected by resource depletion and are allowed to make further investment in the purpose of gaining additional resources (Hobfoll, 2001; Halbesleben et al., 2014). Taking charge emphasizes constructive challenges to the *status quo*. Individuals should not only take risks but also invest a lot of time and energy when they take charge at work (Mcallister et al., 2007). In addition, employees engaging in taking charge may cause the loss of individual resources, thus they are inclined to take resource protection measures and are unwilling to spend individual resources to take charge. However, for individuals with abundant resources, taking charge is also a method of gaining more resources, and people are more likely to take risky behavior (Hobfoll, 2002).

As a kind of energy resource, thriving not only makes up for the resource loss of employees in the process of taking charge but also improves the employees’ ability to gain more resources (Sonnentag and Fritz, 2007; Yanfei et al., 2020). More specifically, as a positive emotional experience, vitality enables employees to be energetic at work. Employees who experience vitality at work will not limit their efforts to meet the organization’s requirements and goals, but often exceed the formal work requirements and make extra efforts in their work (Kleine et al., 2019). Furthermore, given that taking charge challenges the organizational *status quo*, it is likely to be unpopular with supervisors and colleagues if the organizational changes do not work perfectly from the very beginning, which often involves setbacks and failure (Mackenzie et al., 2011). Vitality can promote employees to broaden their thought—action repertoires and develop positive psychological resources (e.g., optimism and resilience), which is helpful for employees engaging in taking charge, enabling them to be more optimistic about challenges and resistance (Carmeli and Spreitzer, 2009). In addition, learning helps employees to master job-related knowledge and skills, and improved knowledge and skills will further increase their confidence to complete challenging work (Carmeli and Spreitzer, 2009). Scholars have also argued that when employees acquire new knowledge and skills at work, they are likely to proactively go beyond the *status quo* and try new things such as exploring new workflow (Kleine et al., 2019). In sum, we propose the following hypothesis:

Hypothesis 3 (H3). Thriving at work is positively related to taking charge.

Combined with the above analysis, this study suggests that CSR can indirectly promote employees’ taking charge through the mediating role of thriving at work. Enterprises can focus their efforts on CSR and present a capable and responsible corporate image to employees. As a kind of contextual resource, a good corporate image can help employees accumulate resources and improve their experience of vitality. In addition, the implementation of the CSR programs for employees can be regarded as a resource of social support to help an employee to learn at work. On the other hand, according to the resource gain spiral of COR, thriving at work as an energy resource will increase the initial resource accumulation of employees, making them capable of resisting potential resource loss, and actively taking charge to obtain more resources (Halbesleben et al., 2014). In short, the following hypothesis is proposed:

Hypothesis 4 (H4). Thriving at work positively mediates the relationship between CSR and taking charge.

### The Moderating Role of Task Significance

Existing studies show that not all CSR practices can promote employees’ positive cognition and behavior (Vlachos et al., 2017), and an important reason for this is that employee CSR perception is affected by a variety of factors (Gond et al., 2017). As a core work characteristic, task significance reflects the extent to which the work itself influences the life or work of people within and outside the organization (Farooq et al., 2017). Since employees are increasingly concerned with the social value of their work, task significance has gained increasing academic attention in human resource management (Turban and Greening, 1996). Research studies on task significance focus on how individuals’ perception of being able to make a difference to others relates to their work attitudes and performance (Grant, 2007, 2008). Ong et al. (2018) also develop a CSR sensitivity framework to explain how task significance influences the strength of the positive association between CSR and OCB. Given the above, this study argues that task significance may be an important moderating variable between employees’ perception of CSR and thriving at work.

Employees whose jobs are higher in task significance are likely to experience their work as more meaningful—that is, more purposeful and valuable (Hackman and Oldham, 1975, 1976). To have a greater positive impact on others, jobs with high task significance motivate employees to invest more time and energy in their work (Grant, 2008), which will consume more individual resources. According to COR theory, time or energy investment can be regarded as resource loss. Because the return on the resource investment is uncertain, employees are likely to be anxious and stressed (Hobfoll, 1989). Under the circumstances, the implementation of CSR by the organization cannot promote employee thriving effectively. Moreover, since CSR and task significance in promoting employees’ thriving is homogeneous and complementary, the impact of CSR on thriving at work will also be limited when the jobs are higher task significance. To be more specific, CSR is an action taken by an organization to promote social welfare in considering the needs of stakeholders (Waldman et al., 2006; Barnett, 2007), and it meets the employees’ expectations that it should have a positive impact on people within and outside the organization. Employees who work in such a responsible enterprise will feel more meaningful. On the other hand, a high level of task significance also reflects the extent to which the work has an important influence on the employee and the organization. Under the condition of high task significance, both CSR activities and the jobs can give employees a sense of meaning, which can be regarded as the same resources in influencing employees and enabling them to thrive at work (Spreitzer et al., 2005). Therefore, when task significance is high, the role of CSR in promoting employees’ thriving is limited.

On the contrary, when the level of task significance is low, CSR is an important factor in promoting employees’ thriving at work. This is because employees with low task significance usually need to complete auxiliary work and do not play an important role in the organization, and their work has less impact on the organization (Parker, 2014). In this instance, the incentive effect of the work is low, which leads to the perception that work is meaningless. The work being perceived as meaningless will result in difficulties gaining resources from work (Brummelhuis and Bakker, 2012), and employees also lack initiative or positive emotion in their work (Jianghua et al., 2017). In this case, employees will need more motivation from other external sources. If an enterprise actively undertakes CSR, the improvement of the enterprise’s image and social status can be regarded as a resource that helps employees indirectly realize their value (Halbesleben et al., 2014). Taking on social responsibility for employees can also provide opportunities to learn, and create an atmosphere of respect within the organization, which will improve their experiences of vitality and learning (Paterson et al., 2014). Therefore, employees with low task significance are more sensitive to the CSR practice of the organization, and they will show a more positive attitude and behavior in their work under the influence of their employer. The implementation of CSR provides employees with a more positive work experience, which further enables employees to thrive (Spreitzer et al., 2005). In a word, when jobs are lower in task significance, CSR has a significant positive impact on thriving at work. Accordingly, we propose the following hypothesis:

Hypothesis 5 (H5). Task significance negatively moderates the relationship between CSR and thriving at work, which is stronger when task significance is low.

By combining Hypotheses 1, 2, 3, 4, and 5, we constructed a moderated mediation model and proposed that task significance can moderate the indirect effect of CSR perception on employees’ taking charge via thriving at work. To be more specific, when task significance is lower, the impact of CSR perception on employees’ thriving at work will be greater, and the indirect impact of CSR through thriving at work on the employees’ taking charge will be more positive. Conversely, since CSR and task significance in promoting employees’ thriving is homogeneous and complementary, when employees whose jobs are higher in task significance, CSR has a lower level of influence on employee’s taking charge via thriving at work. Therefore, the following hypothesis is proposed:

Hypothesis 6 (H6). Task significance negatively moderates the mediating effect between CSR and taking charge, such that the relationship is more positive for employees who report low task significance than those who report high task significance.

**FIGURE 1:**
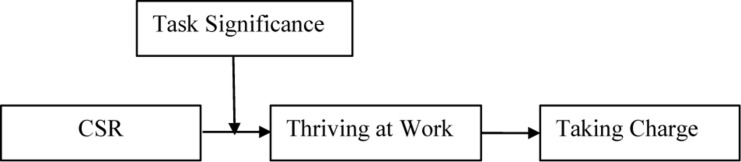
Theoretical model.

The conceptual model for this research is shown in Figure 1.

## Materials and Methods

### Sample and Procedures

Following the suggestions of scholars, we utilized a snowball sampling method to recruit working adult employees to complete an online survey (Ong et al., 2018; Fan et al., 2020). The survey began in March 2020, we first contacted the authors’ MBA students or former classmates who were working in enterprises and asked them to invite their colleagues to participate in this survey. Then we contacted the enterprises that have cooperation with the author and sent the online questionnaire to the person in charge of the human resources department of the enterprise, asking them to send the questionnaire to employees. To improve the response rate of questionnaires, before conducting a formal investigation, we sent a cover letter with detailed information about the purposes of the study, assurances of confidentiality of data, and the personal anonymity of participants, and we also clarified that there is no right and wrong answer to any question and that their response is valuable for us. Moreover, to collecting data more widely and enhance the validity of the survey results, we selected employees from Anhui, Hunan, Zhejiang, Shandong province, and other provinces in China for the survey, including industries such as real estate, internet, communication, manufacturing, service, consumption industry, and so on.

We collected 594 questionnaires in total, and 444 questionnaires were valid, the overall response rate was 74.75%. Among the 444 employees, 43.70% were male and 56.30% were female. A total of 75.70% of employees had a bachelor’s degree. The average age was 28.18 years (*SD* = 6.127). Regarding the position, 64.60% were non-managers, 23.40% were first-line managers, 10.40% were middle managers, and 1.60% were top managers. Finally, the average organizational tenure of respondents was 3.75 years (*SD* = 4.097).

### Measures

The measures we chose were initially written in English, which are mature and reliable. To ensure the reliability and validity of the questionnaire, this study adopted the principle of “translation-back translation,” and a pilot study was conducted with MBA students. The questionnaire was revised repeatedly based on the feedback to accurately reflect the original intention of the developers, and also suit the context of China. In this study, all variables were both conceptualized and measured at the individual-level of analysis, and measures of our focal variables are provided in Appendix.

1. Corporate social responsibility. We used the 5-item CSR scale developed by Rupp et al. (2018), a sample item including “Flexible company policies enable employees to better coordinate work and personal life.” Response options ranged from 1 (strongly disagree) to 7 (strongly agree). Higher scores are indicative of employees perceiving more CSR activity by their employer. The scale’s Cronbach’s α value is 0.823 in this study.
2. Thriving at work. Thriving at work was measured by using the 10-item scale developed by Porath et al. (2012), which assesses the two subdimensions of employees’ thriving. A sample item includes “At work, I find myself learning often.” Response options ranged from 1 (strongly disagree) to 7 (strongly agree). Furthermore, according to previous empirical studies on thriving at work, although it has two dimensions, scholars usually calculate the average score for subsequent analysis (Hildenbrand et al., 2018; Lin et al., 2020). The scale’s Cronbach’s α value was 0.847 in this study.
3. Task significance. The significance of work is measured by using the task significance scale in the WDQ scale compiled by Morgeson and Humphrey (2006), There are four items in total, including “The results of my work are likely to significantly affect the lives of other people.” Using the Likert 5-point scale, response options ranged from 1 (strongly disagree) to 5 (strongly agree), and the Cronbach’s α value is 0.802 in this study.
4. Taking Charge. We adopted the scale modified by Ruoyong et al. (2018) according to Morrison and Phelps (1999) research assessing employees’ taking charge. There was a total of 10 items, with example items including statements such as “In my work, I often try to adopt improved procedures for doing my job,” using the Likert 5-point scale, the response options ranged from 1 (strongly disagree) to 5 (strongly agree). The Cronbach’s α value was 0.895 in this study.
5. Control variables. Consistent with prior research on taking charge, employees’ age was related to taking charge because it is associated with uncertainty and risk (Li et al., 2016). Scholars also suggest that tenure and position in the organization have a significant impact on employees’ taking charge (Morrison and Phelps, 1999). Accordingly, we controlled for multiple factors that might impact our results, including employees’ age, tenure and position (1 = non-managers, 2 = first-line managers, 3 = middle managers, 4 = top managers), age and tenure in the enterprise was measured using the respondents’ self-reported years.

### Common Method Bias Test

This study controls the common method deviation from two aspects: procedural control and statistical testing (Podsakoff et al., 2003). Procedural control includes the methods of reverse scoring for some questionnaire items, separating the arrangement of different scales, and allowing participants to answer the questionnaires anonymously. In addition, we used the Harman single factor in SPSS25.0 statistical analysis software to test whether the common methods variance is in the acceptable range. The results show that the first factor can explain 33.621% of variances, which is far below 40%, and it shows that the CMV of the data is not significant and will not affect the reliability of the research conclusion.

### Confirmatory Factor Analysis

To verify the validity of these constructs in the present study, we conducted a CFA by using AMOS26.0. As shown in Table 1, the four-factor model (χ^2^/df = 1.848, CFI = 0.960, TLI = 0.951, IFI = 0.961, RMSEA = 0.044) is statistically superior to the single-factor, two-factor and three-factor models. Therefore, the four-factor model is more appropriate. The above results revealed that the model we proposed has the best validity.

**TABLE 1:** Results of confirmatory factor analysis of the measurement models.

Models	χ^2^/df	CFI	TLI	IFI	RMSEA
4-factor model (CSR; TW; TS; TC)	1.848	0.960	0.951	0.961	0.044
3-factor model (CSR + TW; TS; TC)	3.720	0.857	0.842	0.858	0.078
2-factor model (CSR + TW + TS; TC)	4.862	0.794	0.776	0.795	0.093
1-factor model (CSR + TW + TS + TC)	8.500	0.596	0.564	0.598	0.130

**N = 444; CSR represents Corporate Social Responsibility; TW represents thriving at work; TS represents task significance; TC represents employees’ taking charge. CFI is the Comparative Fit Index; TLI is the Tucker-Lewis Index; IFI is the Incremental Fit Index; RMSEA is the Root-Mean-Square Error of Approximation.**

## Results

### Descriptive Statistical Analysis

Table 2 shows the means, standard deviations, and Spearman’s correlation coefficients of all constructs used in the conceptual model. The results revealed that CSR is strongly related to employees’ thriving at work (*r* = 0.572, *p* < 0.01) and taking charge (*r* = 0.286, *p* < 0.01); thriving at work and taking charge (*r* = 0.476, *p* < 0.01) also had a positive correlation. Correlation analysis provides a premise for further study of the relationship between variables.

**TABLE 2:** Means, standard deviations, and correlations among variables.

Variables	Mean	SD	1	2	3	4	5	6	7	8	9
1. Gender	–	–	–								
2. Education	–	–	0.083	–							
3. Position	–	–	−0.211**	0.075	–						
4. Age	28.182	6.127	−0.108*	0.031	0.396**	–					
5. Tenure	3.752	4.097	–0.053	−0.102*	0.310**	0.469**	–				
6. CSR	5.333	1.046	0.089	0.027	0.014	0.096*	0.041	–			
7. TW	5.240	0.852	0.033	0.055	0.182**	0.222**	0.101*	0.572**	–		
8. TS	3.448	0.649	–0.092	0.140**	0.082	0.070	0.010	0.325**	0.401**	–	
9. TC	3.622	0.590	–0.038	0.030	0.272**	0.251**	0.175**	0.286**	0.476**	0.435**	–

N = 444; CSR represents Corporate Social Responsibility; TW represents thriving at work; TS represents task significance; TC represents employees’ taking charge.*p < 0.05; **p < 0.01; ***p < 0.001.

### Hypothesis Testing

Before hypothesis testing, we first used SPSS25.0 software to test the multicollinearity of data. The variance inflation factors (VIF) for CSR perceptions (1.512), thriving at work (1.612), and task significance (1.211) were less than 2, far below the generally accepted 10, which revealed that multicollinearity was not a concern.

The statistical validity of the hierarchical regression method has been questioned by many scholars in recent years, this study used the PROCESS SPSS macro developed by Hayes (Hayes and Scharkow, 2013) to test the hypothesis, which allows estimating simultaneously indirect and moderated effects, and moderated regression analysis. Furthermore, we set the number of Bootstrap samples to 5,000 and selected the 95% confidence interval when testing the hypothesis.

1. The main effect test of CSR on taking charge. We first ran a mediation model (Process Model 4) to test the main effect of CSR perception on taking charge. After controlling the variables that are significantly related to taking charge, such as employee age, tenure, and position, the results of model 1 in Table 3 indicate that CSR has a significant positive effect on employees’ taking charge (β = 0.152, *p* < 0.001, 95% CI [0.104, 0.201]), the 95% CI excludes zero. Therefore, Hypothesis 1 is supported.

**TABLE 3:** Main effect and mediating effect analysis results.

Variables	Taking charge		Thriving at work		Taking charge	
	Model 1		Model 2		Model 3	
	b	SE	b	SE	b	SE
**Control variables**						
Age	0.012*	0.005	0.016**	0.006	0.007	0.005
Tenure	0.006	0.007	–0.002	0.009	0.007	0.007
Position	0.164***	0.038	0.139**	0.046	0.122***	0.036
**Independent variables**						
CSR	0.152***	0.025	0.381***	0.032	0.026	0.028
Mediating variable						
Thriving at Work					0.277***	0.036
**Moderating variable**						
Task significance			0.273***	0.051		
CSR* Task significance			−0.121**	0.041		
R2	0.172		0.427		0.273	
F	22.800***		54.216***		32.832***	

**Direct effect of CSR on taking charge**	**Effects**		**SE**	**LLCI**	**ULCI**	

	0.026		0.028	–0.029	0.082	

*p < 0.05; **p < 0.01; ***p < 0.001.

**FIGURE 2:**
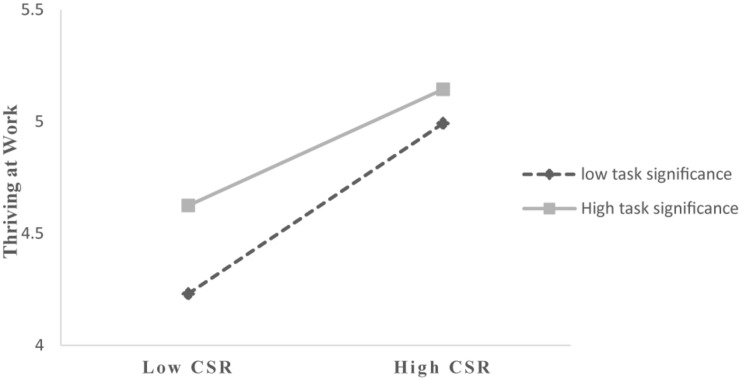
Moderating effect diagram of task significance.

2. The mediating effect test of thriving at work. We then used a moderated mediation model (Process Model 7) to test the mediating effect of thriving at work. As shown in Model 2 and Model 3 in Table 3, when controlling employees’ age, tenure and position, CSR has a significant positive impact on employees’ thriving at work (β = 0.381, *p* < 0.001, 95% CI [0.318, 0.443]), thus Hypothesis 2 is supported. In support of Hypothesis 3, the results confirmed that thriving at work is positively related to employees’ taking charge (β = 0.277, *p* < 0.001, 95% CI [0.207, 0.347]), the results supported Hypothesis 3. After controlling the mediating variable (thriving at work), the direct effect of CSR on employees’ taking charge is 0.026 and the 95% confidence interval is [-0.029, 0.082], including 0. Combined with the results of the main effect test, thriving at work completely mediates the relationship between CSR and employees’ taking charge, and Hypothesis 4 is supported.
3. The moderating effect test of task significance. MODEL 7 in PROCESS SPSS Macro continues to be used to test the moderated mediating hypothesis. The results of the moderating effect analysis are shown in Model 2 in Table 3. From the results, we can see that the interaction of CSR and task significance has a significant impact on thriving at work (β = -0.121, *p* < 0.001), 95% CI [-0.201, -0.041], excluding 0, indicating that task significance negatively moderates the relationship between CSR and employees’ thriving at work, thus hypothesis 5 is supported. To assess whether the moderating effect was consistent with the hypothesis, the relationship between thriving at work and employees’ CSR perception is plotted in Figure 2 (Preacher and Hayes, 2004). The results of a simple slope test indicate that compared with the case of high levels of task significance, the positive effect of CSR on employees’ thriving at work is stronger in the case of the jobs that are lower in task significance. That is to say, task significance will weaken the positive effect of CSR on thriving at work, and the results further demonstrate the negatively moderating role of task significance.

4. Moderated mediating effects tests. The results in Table 4 show that in the case of low levels of task significance (mean minus 1 standard deviation), the interaction effect of CSR perception and the task significance on employees’ taking charge via thriving at work is significant (95% CI [0.087, 0.177]); when task significance was high (mean plus 1 standard deviation), although the indirect effect of CSR on taking charge through thriving at work is also significant (95% CI [0.050, 0.122]). The indirect effect was smaller than that in the jobs that are lower in task significance. The moderated mediating index value is -0.034, and the confidence interval is [-0.072, -0.003]. The above results reveal that task significance negatively moderates the indirect effect of CSR on employees’ taking charge via thriving at work. Therefore, Hypothesis 6 is supported.

**TABLE 4:** Results of moderated mediating effect analysis.

Mediating variable	Task significance	Effect	BootSE	BootLLCI	BootULCI
Thriving at work	Low	0.127	0.023	0.087	0.177
	Medium	0.105	0.017	0.073	0.141
	High	0.084	0.019	0.050	0.122
The moderated mediating effect index		–0.034	0.018	–0.072	–0.003

## Conclusion

Based on COR theory, this study discussed the influence mechanism of CSR on employees’ taking charge. The findings demonstrate that employees’ CSR perception can positively influence them taking charge and that thriving at work acted as a mediator for this link. Moreover, we find that task significance negatively moderated the relationship between employees’ CSR perception and taking charge via thriving at work. That is to say, employees’ CSR perception and task significance interact to predict employees’ taking charge via thriving at work, such that an organization’s CSR is more positively associated with taking charge among employees who report lower task significance than among those who report higher task significance.

Our results are consistent with COR theory, which suggest that individuals with abundant resources are not easily affected by resource depletion and are more capable of acquiring more resources to realize the spiral of resource appreciation (Hobfoll, 2002). As a kind of contextual resource and social support, enterprise actively undertaking CSR promotes employees’ taking charge via thriving at work. This positive relationship is also supported by Zeng et al. (2020a), who suggests mentoring as a resource that has a significant positive impact on protégés’ adaptive performance via thriving at work. Furthermore, findings confirm the importance of CSR and its boundary context to employees’ taking charge, which provides a very promising framework for studying CSR and taking charge behavior via thriving at work.

## Discussion

### Theoretical Contribution

First, unlike previous micro-level CSR studies focused on affiliative organizational citizenship behaviors, this study argues that employees’ taking charge is a type of challenging organizational citizenship behavior characterized as risky and constructive. Exploring the relationship between CSR perception and employees’ taking charge can further broaden literature on the dependent variables of CSR (Rupp and Mallory, 2015). Furthermore, the result that CSR positively affects employees’ taking charge also supports previous literature that CSR can promote risky and constructive behaviors in employees, such as voice behavior and innovation behavior (Glavas, 2016; Juan et al., 2019).

Second, by adopting COR theory as a theoretical foundation, we introduced thriving at work as an individual energy resource and that an enterprise that positively undertakes responsibility can use it as a social support and contextual resources in understanding the potential impact of CSR on employees’ taking charge. Our findings suggest that employees’ CSR perception positively influences taking charge via thriving at work. These findings support the resource gain spiral of COR theory that individuals with abundant resources are in a better position to invest and gain additional resources (Halbesleben et al., 2014). It not only helps us gain more insight into the underlying mechanism of CSR influencing employees’ taking charge, but also promotes the development of COR theory. Since thriving at work is a relatively new construct that has not yet received much research attention (Kleine et al., 2019), this study also contributes to research on the antecedents and outcome variables of employees’ thriving, and supports the view of previous scholars that thriving at work is regarded as an energy resource that can promote positive behaviors in employees (Jie et al., 2019).

Third, scholars began to emphasize the importance of examining the boundary conditions of micro-level CSR (Gond et al., 2017). Existing research investigating the moderating mechanism of CSR mainly focuses on personal characteristics and cultural differences, and conditional factors such as job characteristics are ignored (Vlachos et al., 2017). Therefore, this research adopts task significance as a moderating variable from the perspective of job characteristics, which is also unique in identifying boundary conditions for the relationship between CSR and employees’ taking charge. The empirical study shows that task significance, as an important contextual factor for employees’ perception of the meaning of work, is similar to the meaning perceived by employees when their employer positively undertakes CSR practices. Moreover, to better accomplish important tasks, jobs with higher task significance objectively require employees to invest more time and energy in their work, which may force employees to consume more individual resources. The consumption of resources may bring anxiety and pressure to employees. Therefore, for employees whose jobs are high in task significance, the role of CSR in promoting employees’ taking charge through thriving is limited. However, for those whose jobs are low in task significance, CSR can make up for a lack of meaning at work, which will further promote employees to thrive and effectively motivate employees to take charge.

### Practical Implications

In an increasingly complex and changeable business environment, enterprises must take into account social responsibilities and encourage employees to make efforts for taking charge in the organization, which will ensure organizational sustainability. This empirical study has the following three management implications:

First of all, from a practical perspective, this research shows that employees’ perceptions of CSR practices can pay off in terms of increased thriving at work and taking charge. This suggests that organizations should make efforts to publicize CSR programs (both formally and informally). In other words, the enterprise should have a humanitarian spirit and take into account the expectations of the public, and actively implement practices and activities that can promote social public welfare such as making charitable donations and developing programs for environmental protection. Additionally, entrepreneurs and managers should view employees as important stakeholders and respect their contribution to daily management. In particular, enterprises should provide flexible working conditions and express care and kindness by adopting policies to help employees achieve professional development, which can further motivate employees to take charge.

Secondly, entrepreneurs and managers need to make policies in daily management to increase employees’ experience of thriving. To be more specific, when taking relevant policies, entrepreneurs and managers must consider employees’ basic psychological needs, such as needs for autonomy and competence to improve their thriving at work. Moreover, enterprises can also provide employees with the opportunity to learn, to increase their knowledge and skills through training activities, and to create a suitable working environment for them to apply learned knowledge and skills. To further increase employees’ experience of thriving at work, entrepreneurs and managers need to pay attention to employees’ emotional states at work and express organizational trust and respect to employees.

Finally, entrepreneurs and managers should improve a sense of meaning in employees whose jobs are low in task importance. Research findings reveal that for employees with lower task significance, CSR has a stronger effect on their thriving at work, and the experience of thriving will further encourage employees to take charge. Therefore, for employees who are engaged in a low level of task significance, enterprises should appropriately take part in CSR activities or use other measures to evoke their vitality and learning, to change employees’ psychological states at work. More specifically, to improve the level of thriving at work, enterprises can take measures such as offering full authorization, enriching work content, and strengthening communication at work. Moreover, managers can also put rewards and incentives in place to encourage employees to change the boundaries of their jobs and make their work more meaningful.

### Limitations and Future Directions

Although our research has certain theoretical contributions and practical implications, we admit that there are still some research limitations. Firstly, CSR can be divided into internal CSR for employees and external CSR for external stakeholders. The influence of internal CSR and external CSR on employees’ taking charge may be different, future research can consider the effect of the specific dimensions of CSR on employees’ taking charge behavior. Moreover, Previous research shows that individuals from different cultures may respond to CSR differently because they hold dissimilar values (Aguinis and Glavas, 2017). Since we conducted our research in China, it is not clear whether the data might have been biased due to the unique cultural context. Therefore, future research must test how cultural values affect employees’ reactions to internal or external CSR.

Secondly, from the perspective of job design, this study examines the moderating role of task significance for the effect of CSR perception on employees’ taking charge via thriving at work. Future research may consider other job characteristics (e.g., job autonomy and job integrity) and leadership styles (e.g., responsible leadership) to further expand the research of micro-level CSR. Moreover, based on COR theory, we argue that thriving at work plays a mediating role for CSR and employees’ taking charge. Future research needs to explore other underlying mechanisms and the microfoundations of CSR from other theoretical perspectives.

Third, we collected data from self-reports by employees at a single time point. As mentioned previously, we addressed the potential problem regarding common method variance from procedural control and statistical testing. Although there is no serious common method deviation, the impact of common method deviation cannot be eliminated. Future research can collect data from multiple sources and multiple time points or adopt methods of situational experiments to reduce common method variance. Furthermore, existing research shows that individual demographics such as gender, age, education level, tenure, and position can be significantly influenced employees’ taking charge (Morrison and Phelps, 1999; Li et al., 2016). Future research should explore the potential mechanism of the demographic factors mentioned above on employees’ taking charge behavior.

## Data Availability Statement

All datasets generated for this study are included in the article/supplementary material, further inquiries can be directed to the corresponding author/s.

## Author Contributions

AY, LT, and YH contributed to the conception and design of the study. AY provided financial support and research assistance. LT and YH designed the research, collected and analyzed the data, and wrote the first draft of the manuscript. All authors contributed to manuscript revision and approved the submitted version.

## Conflict of Interest

The authors declare that the research was conducted in the absence of any commercial or financial relationships that could be construed as a potential conflict of interest.

## Appendix

### CSR Perception (Rupp et al., 2018)

1. Our business supports employees’ education.
2. Flexible company policies enable employees to better coordinate work and personal life.
3. Our business gives adequate contributions to charities.
4. A program is in place to reduce the amount of energy and materials wasted in our business.
5. We encourage partnerships with local businesses and schools.

### Thriving at Work (Porath et al., 2012)

**(1) Learning latent factor**

1. At work, I find myself learning often.
2. At work, I continue to learn more and more as time goes by.
3. At work, I see myself continually improving.
4. At work, I am not learning (R).
5. At work, I have developed a lot as a person.

**(2) Vitality latent factor**

1. At work, I feel alive and vital.
2. At work, I have energy and spirit.
3. At work, I do not feel very energetic (R).
4. At work, I feel alert and awake.
5. At work, I am looking forward to each new day.

### Task Significance (Morgeson and Humphrey, 2006)

1. The results of my work are likely to significantly affect the lives of other people.
2. The job itself is very significant and important in the broader scheme of things.
3. The job has a large impact on people outside the organization.
4. The work performed on the job has a significant impact on people outside the organization.

### Taking Charge (Morrison and Phelps, 1999; Ruoyong et al., 2018)

1. In my work, I often try to adopt improved procedures for doing my job.
2. In my work, I often try to change how my job is executed to be more effective.
3. In my work, I often try to bring about improved procedures for the work unit or department.
4. In my work, I often try to institute new work methods that are more effective for the company.
5. In my work, I often try to change organizational rules or policies that are non-productive or counterproductive.
6. In my work, I often make constructive suggestions for improving how things operate within the organization.
7. In my work, I often try to correct a faulty procedure or practice.
8. In my work, I often try to eliminate redundant or unnecessary procedures.
9. In my work, I often try to implement solutions to pressing organizational problems.
10. In my work, I often try to introduce new structures, technologies, or approaches to improve efficiency.
